# UBAP2L promotes gastric cancer metastasis by activating NF-κB through PI3K/AKT pathway

**DOI:** 10.1038/s41420-022-00916-7

**Published:** 2022-03-19

**Authors:** Ou Li, Cheng Zhao, Jian Zhang, Feng-Nan Li, Zi-Yi Yang, Shi-Lei Liu, Chen Cai, Zi-Yao Jia, Wei Gong, Yi-Jun Shu, Ping Dong

**Affiliations:** 1 Laboratory of General Surgery and Department of General Surgery, Xinhua Hospital affiliated with Shanghai Jiao Tong University School of Medicine, No. 1665 Kongjiang Road, 200092 Shanghai, China; 2 Shanghai Key Laboratory of Biliary Tract Disease Research, No. 1665 Kongjiang Road, 200092 Shanghai, China

**Keywords:** Gastric cancer, Gastric cancer

## Abstract

Ubiquitin-associated protein 2-like (UBAP2L) is highly expressed in various types of tumors and has been shown to participate in tumor growth and metastasis; however, its role in gastric cancer (GC) remains unknown. In this study, we observed that UBAP2L expression was markedly elevated in GC tissues and five GC cell lines. Higher expression of UBAP2L was associated with poor prognosis as revealed by bioinformatics analysis on online websites and laboratory experiments. Knockdown of UBAP2L impeded the migration and invasion abilities of GC cell lines. In contrast, its overexpression enhanced the migration and invasion abilities of GC cell lines. Overexpression of UBAP2L also increased the number and size of lung metastatic nodules in vivo. According to the results of mass spectrometry and pathway annotation of the identified proteins, the PI3K/AKT pathway was found to be related to UBAP2L regulation. Further exploration and rescue experiments revealed that UBAP2L stimulates the expression and nuclear aggregation of p65 and promotes the expression of SP1 by activating the PI3K/AKT pathway. In summary, our findings indicate that UBAP2L regulates GC metastasis through the PI3K/AKT/SP1/NF-κB axis. Thus, targeting UBAP2L may be a potential therapeutic strategy for GC.

## Introduction

Gastric cancer (GC) is one of the most prevalent types of cancer worldwide, contributing to over one million cases and an estimated 769 000 deaths in 2020, ranking fifth in incidence and fourth in mortality. According to the Global Burden of Disease Study 2019, disability-adjusted life years in China account for 44.21% of the total number of GC cases worldwide [1]. Despite the advances made in the multimodal treatment of GC, recurrences are common [2]. Therefore, it is imperative to explore the underlying mechanisms of GC development and identify new treatments to improve the prognosis of GC.

Ubiquitin-associated protein 2-like (UBAP2L), which is also referred to as NICE-4, has a ubiquitin-associated domain and multiple RGG/RG repeats at the N-terminus [3]. Long-term studies have revealed its various functions. UBAP2L interacts with human zona pellucida3 (ZP3) [4], co-fractionates with ubiquitin in the high-density fraction, and colocalizes in the ubiquitin-containing aggregates after proteasome inhibition [5]. UBAP2L is part of a PcG subcomplex comprising BMI1 which is essential for the activity of LT-HSCs [6]. Arginine methylation in UBAP2L is required for the accurate distribution of chromosomes during mitosis [3] and modulates stress granule assembly [7].

Recent studies have illustrated that UBAP2L participates in the growth and metastasis of various types of cancers, such as prostate cancer [8], colorectal carcinoma [9], hepatocellular carcinoma [10–12], glioma [13], lung adenocarcinoma [14], and breast carcinoma [15]. However, its role in the development and progression of GC remains unclear. In the present study, we evaluated the expression of UBAP2L using bioinformatics databases, GC cell lines, and tissue specimens. The suppression of UBAP2L expression reduced the migration and invasion abilities of GC cell lines. A preliminary investigation of the mechanism was conducted. The mass spectrometry results predicted that UBAP2L may be associated with the PI3K/AKT pathway. Further exploration verified the assumption that UBAP2L works by activating the PI3K/AKT/SP1/NF-κB axis. Subsequently, we performed rescue assays and in vivo mouse lung metastases to further confirm its function.

## Results

### Expression of UBAP2L is elevated in GC tissues and cell lines

**Fig. 1:**
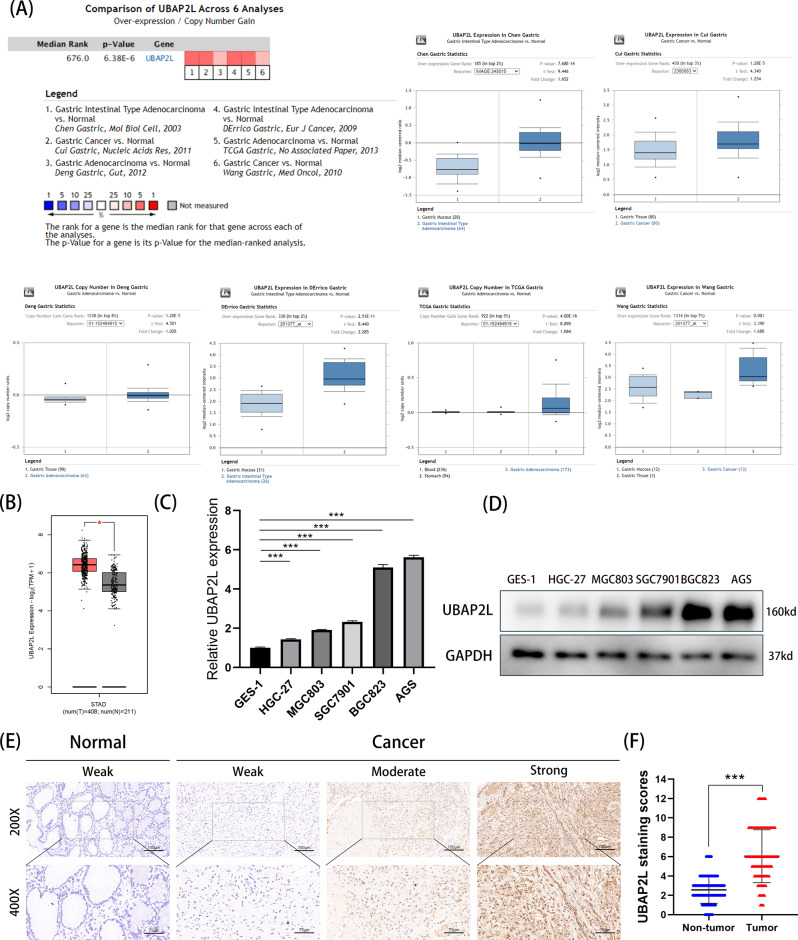
UBAP2L is upregulated in gastric cancer. **A** Based on the Oncomine database, UBAP2L mRNA overexpression showed statistically significant in 6 datasets (red). **B** Relative UBAP2L expression in GC and adjacent normal tissue based on TCGA database. **C**, **D** Relative UBAP2L mRNA and protein expression in HGC-27, MGC03, SGC7901, BGC823, AGS, and GES-1 detected by quantitative real-time PCR and western blot. **E** Representative images of immunohistochemical staining for UBAP2L in GC tissue and adjacent normal tissues. **F** The score of immunohistochemical staining in 150 paired tumor and nontumor.

To identify the expression pattern of UBAP2L in GC, we first investigated the Oncomine database and found that the expression of UBAP2L in GC tissues was significantly higher than that in matched adjacent normal tissues in six analyses (Fig. 1A). Consistent with the results of the Oncomine database, the expression of UBAP2L was upregulated in GC according to the GEPIA2 website, which is based on the Cancer Genome Atlas (TCGA) database (Fig. 1B). Additionally, one gastric mucosal epithelial cell line (GES-1) and five GC cell lines (HGC-27, MGC803, SGC7901, BGC823, and AGS) were used to verify the expression of UBAP2L using qRT-PCR and western blotting. Consistent with the result of the aforementioned databases, the expression of UBAP2L in the five GC cell lines (Fig. 1C, D) was much higher than that of GES-1. We also assessed the expression of UBAP2L in 150 GC tissues and adjacent normal mucosa by immunohistochemistry. Among 150 GC tissues, 107 patients were scored under the high expression group, whereas only 8 patients scored under the high expression group in the normal tissue (Fig. 1E, F). These results suggest that UBAP2L expression is much higher in GC tissues than in non-tumor tissues.

### The relationship between UBAP2L and clinicopathological parameters of GC

As shown in Table 1, higher expression of UBAP2L in gastric tissues was related to T classification, lymph node metastasis, and TNM stage. However, there was no significant difference between other clinical features and UBAP2L expression.

**Table 1:** Association of UBAP2L expression with clinicopathologic parameters of gastric cancer (GC) patients.

Clinic characteristics	Total	UBAP2L expression		*χ*^2^	*p* Value
	*N* = 150	Low (*n* = 43)	High (*n* = 107)		
Gender					
Male	79	23	56	0.016	0.898
Female	71	20	51		
Age, years					
≤60	37	13	24	1.005	0.316
>60	113	30	83		
Tumor location					
Upper stomach	7	3	4	7.450	0.059
Middle stomach	37	15	22		
Lower stomach	95	20	75		
Mixed	11	5	6		
Borrmann type					
Early stage	10	3	7	4.245	0.120
I + II	45	18	27		
III + IV	95	22	73		
Differentiation level					
Well or moderate	68	22	46	0.827	0.363
Poor or undifferentiated	82	21	61		
T classification					
Tis+T1+T2	55	30	25	**28.441**	**<0.001**
T3+T4	95	13	82		
Lymph node metastasis					
N0	40	19	21	**9.461**	**0.002**
N1–N3	110	24	86		
TNM stage (AJCC)					
0–II	76	32	44	**13.605**	**<0.001**
III+IV	74	11	63		

*AJCC* American Joint Committee on Cancer.

Bold values indicate statistical difference.

**Fig. 2:**
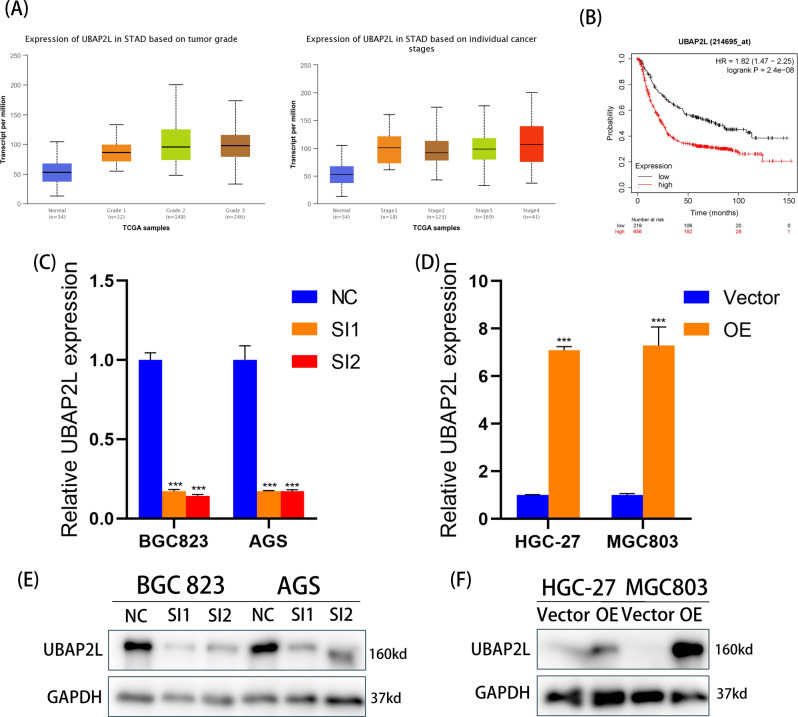
Relationship between UBAP2L and prognosis and validation of knockdown and overexpression. **A** The expression of UBAP2L in individual tumor grades and GC stages based on UALCAN database. **B** Overall survival (OS) of GC patients based on UBAP2L expression using Kaplan–Meier Plotter website. **C**–**F** The efficiency of UBAP2L silence in BGC823 and AGS cell lines and overexpression in HGC-27 and MGC803 cell lines.

We also used the UALCAN database (http://ualcan.path.uab.edu/) to explore the relationship between the mRNA level of UBAP2L and cancer stage, as well as tumor grade. As shown in Fig. 2A, the GC group showed higher UBAP2L expression than the healthy control.

Simultaneously, the Kaplan–Meier plotter database was used to further detect whether UBAP2L mRNA expression influences the prognosis of GC patients. The results revealed that patients with higher expression of UBAP2L had a worse prognosis than those with lower expression (Fig. 2B). Higher expression of UBAP2L was significantly associated with shorter OS in all GC patients (hazard ratio = 1.82; 95% confidence interval, 1.47–2.25; *p* = 2.4e−8).

### Higher expression of UBAP2L induces migration and invasion of GC cells via epithelial-to-mesenchymal transition (EMT)

**Fig. 3:**
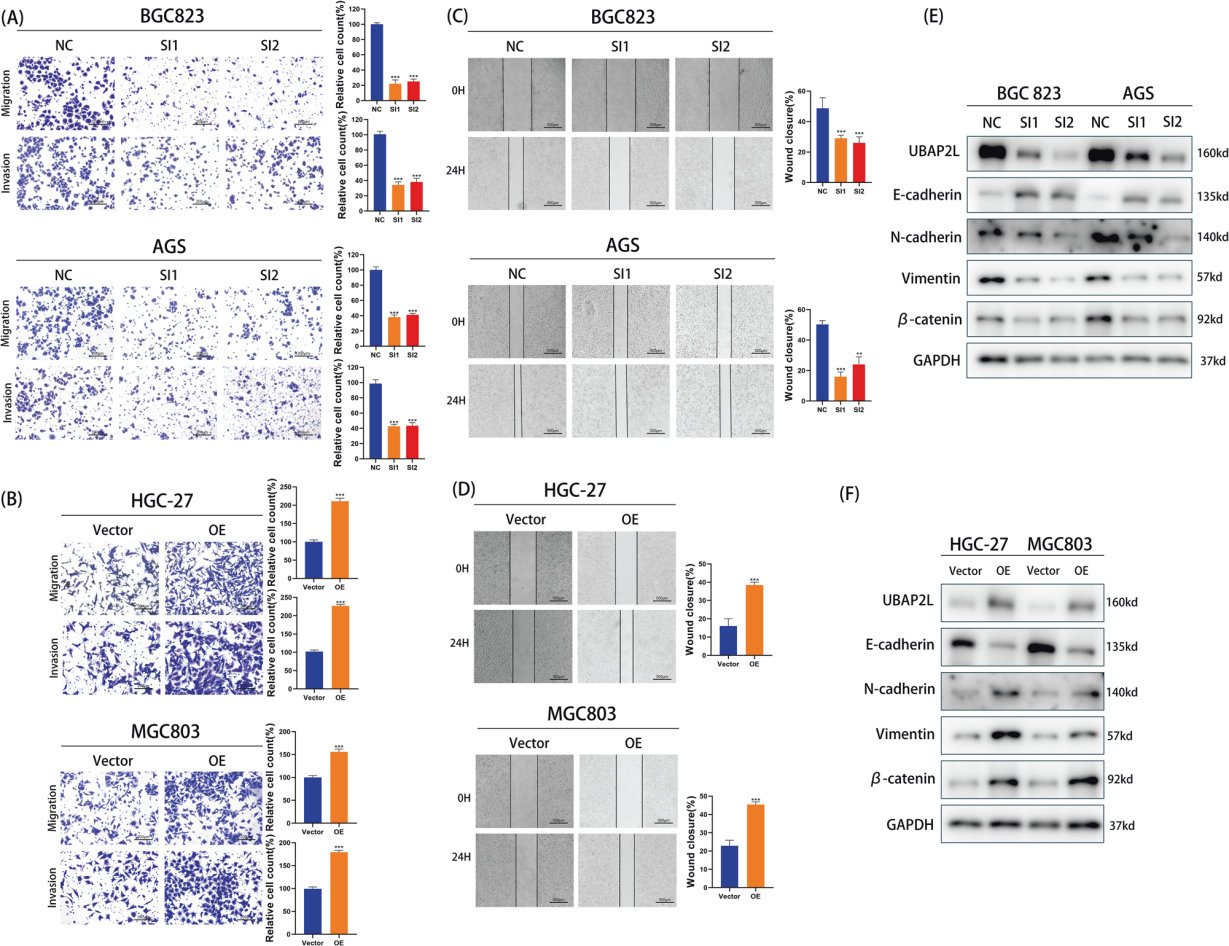
UBAP2L promotes gastric cancer migration and invasion via EMT. **A**, **B** Migrative and invasive capabilities of GC cell lines with UBAP2L knockdown or overexpression was measured by transwell assays. The histogram of relative cell count was exhibited on the right. **C**, **D** Wound healing assay was conducted to declare the migration abilities of GC cell lines. The histogram of the percentage of relative wound closure was exhibited on the right. **E**, **F** Western blot analyzed the expression of E-cadherin, N-cadherin, Vimentin, and β-catenin in GC cells with different UBAP2L expression.

As shown in Fig. 1C, D, expression of UBAP2L was relatively high in BGC832 and AGS cell lines, and relatively low in HGC-27 and MGC803 cell lines. Therefore, we knocked down the expression of UBAP2L in BGC823 and AGS cell lines and overexpressed it in HGC-27 and MGC803 cell lines. To avoid off-target effects, three UBAP2L-specific siRNAs were used to construct UBAP2L knockdown cells and filtered two sequences that had better knockdown efficiency for subsequent studies. Validation of knockout and overexpression efficiency is shown in Fig. 2C–F. We first investigated the effects of UBAP2L knockdown on cell viability; however, there was no significant difference between the NC and SI groups (Supplementary Fig. 1). Further, we confirmed the migration and invasion abilities of GC cell lines using Transwell assays. After the expression of UBAP2L was knocked down by siRNA, the migratory and invasive capabilities of BGC823 and AGS cells decreased significantly compared with that of the negative control cells (Fig. 3A). In contrast, the UBAP2L overexpression in lentivirus-transfected HGC-27 and MGC803 cells showed remarkably enhanced invasion and migration abilities (Fig. 3B). We also carried out a wound-healing assay to further determine the wound healing effect of UBAP2L on GC cells. As shown in Fig. 3C, D, knockdown of UBAP2L considerably suppressed the percentage of wound healing. However, UBAP2L overexpression showed an opposite trend. These results suggest that UBAP2L promotes GC cell migration and invasion in vitro.

EMT has been shown to be associated with the invasive and metastatic phenotype of cancer [16]. To determine whether UBAP2L enhances the migratory and invasive capabilities of GC cell lines via EMT, we determined the expression of EMT biomarkers using western blotting. Knockdown of UBAP2L expression remarkably attenuated the expression of mesenchymal phenotype biomarkers, such as N-cadherin, Vimentin, and β-catenin. In contrast, E-cadherin, which is regarded as a characteristic factor of epithelial cells, was highly expressed (Fig. 3E). However, in UBAP2L-transfected HGC-27, and MGC0803 cells, noticeably higher levels of N-cadherin, Vimentin, and β-catenin and lower levels of E-cadherin were observed (Fig. 3F). These findings indicate that UBAP2L induces migration and invasion of GC via EMT.

### UBAP2L accelerates cell migration and invasion by activating p65 nuclear translocation

**Fig. 4:**
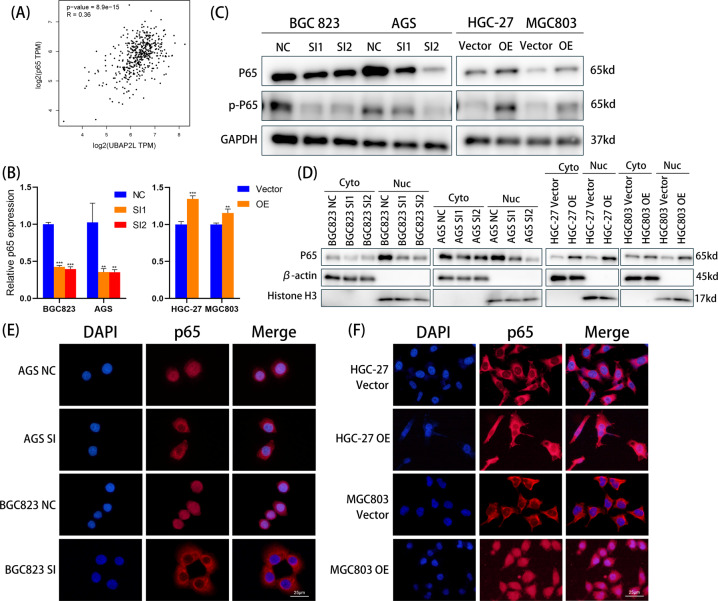
UBAP2L activates p65 expression and facilitate its nuclear translocation. **A** The relationship of mRNA expression between UBAP2L and p65. **B** The mRNA level of p65 detected by quantitative real-time PCR. C The total protein of p65 and pp65 were detected by western blot. **D** The nuclear and cytoplasmic protein extraction of p65 was analyzed by western blot. **E**, **F** The expression and nuclear aggregation of p65 assessed by IF assay.

NF-κB represents a group of structurally related and evolutionarily conserved proteins, with five members in mammals: Rel (c-Rel), RelA (p65), RelB, NF-κB1 (p50 and its precursor p105), and NF-κB2 (p52 and its precursor p100) [17]. Numerous studies have indicated that NF-κB might regulate tumor invasiveness [18], and p65, a subunit of NF-κB, plays an increasing role in promoting cancer metastasis by inducing EMT [19]. However, the relationship between UBAP2L and NF-κB has not yet been identified. Hence, we first analyzed the correlation of UBAP2L mRNA levels with p65 mRNA levels using the TCGA database. As shown in Fig. 4A, UBAP2L mRNA levels showed a positive correlation with p65 mRNA levels as determined using Pearson’s correlation analysis (*R* = 0.36, *p* < 0.001). Further, we investigated the regulatory effect of UBAP2L on the activity of p65 in GC cells. mRNA levels of p65 decreased significantly in BGC823 and AGS cells when UBAP2L was knocked down. However, p65 mRNA levels were elevated in HGC-27 and MGC803 cells transfected with UBAP2L (Fig. 4B). Then, we verified the changes in total p65 protein levels and phosphorylation of p65 (p-p65) in GC cells using western blotting. When UBAP2L was overexpressed, the levels of p65 and p-p65 were remarkably upregulated; however, when the endogenous silencing of UBAP2L was performed, the levels of p65 and p-p65 were remarkably reduced (Fig. 4C), suggesting that the expression and activation of p65 may be regulated by UBAP2L. Furthermore, in BGC823 and AGS cells with UBAP2L knockdown, the reduction in nuclear accumulation of p65 was another demonstration of the decreased activation of NF-κB. In contrast, enhanced activation of NF-κB was marked by an increase in nuclear p65 protein levels in UBAP2L-overexpressing HGC-27 and MGC803 cells (Fig. 4D). The results of IF assay further indicated that the expression of UBAP2L is correlated with the nuclear accumulation of p65 in GC cell lines (Fig. 4E, F).

**Fig. 5:**
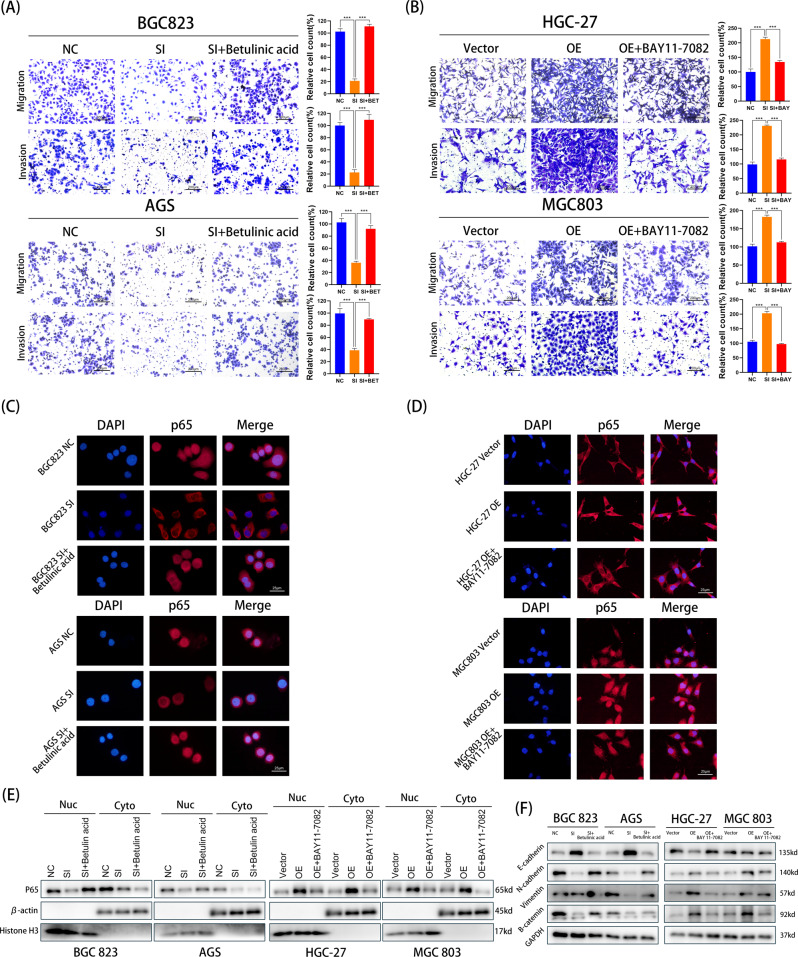
UBAP2L promotes gastric cancer metastasis though p65. **A** Transwell assay demonstrated that Betulinic acid treatment restored the migration and invasion ability of GC cells damaged by siRNA. B BAY11-7082 notably weakened the UBAP2L-induced upregulation in the ability of migration and invasion. **C**, **D** IF displayed that Betulinic acid reversed the activation of p65 attenuated by UBAP2L knockdown and BAY11-7082 suppressed the activation of p65 evoked by overexpression. **E** The expression of nuclear p65 and cytoplasmic p65 were evaluated by western blot in BGC823 and AGS treated by siRNA plus Betulinic acid as well as HGC-27 and MGC803 infected UBAP2L lentivirus plus BAY11-7082. **F** The variation of EMT markers after Betulinic acid or BAY11-7082 treatment.

To further confirm these results, Betulinic acid (an activator of NF-κB) was added to BGC823 and AGS cells with UBAP2L silencing. BAY 11-7082 (an inhibitor of NF-κB) was co-incubated with HGC-27 and MGC803 cells infected with UBAP2L lentivirus. The phenotypes of UBAP2L knockdown BGC823 and AGS cells were rescued by Betulinic acid, which displayed increased migration and invasion abilities (Fig. 5A). The results of western blotting and IF assay illustrated that Betulinic acid restored the nuclear translocation of p65, which was compromised by UBAP2L knockdown (Fig. 5C, E). In contrast, BAY 11-7082 substantially weakened the migration and invasion abilities of UBAP2L-overexpressed HGC-27 and MGC803 cells (Fig. 5B). Similarly, the upregulation of p65 nuclear accumulation in UBAP2L-overexpressed HGC-27 and MGC803 cells was substantially suppressed by BAY 11-7082 (Fig. 5D, E). Changes in EMT biomarker expression were verified using western blotting. The results showed an increase in EMT biomarker expression except for E-cadherin after Betulinic acid treatment. BAY 11-7082 treatment dramatically suppressed the expression of EMT biomarkers induced by UBAP2L overexpression, except for E-cadherin, which showed an opposite trend. (Fig. 5F). These findings revealed that UBAP2L upregulates the expression and nuclear translocation of p65, and thus promotes metastasis.

### UBAP2L activates p65 by stimulating PI3K/AKT/SP1 axis

**Fig. 6:**
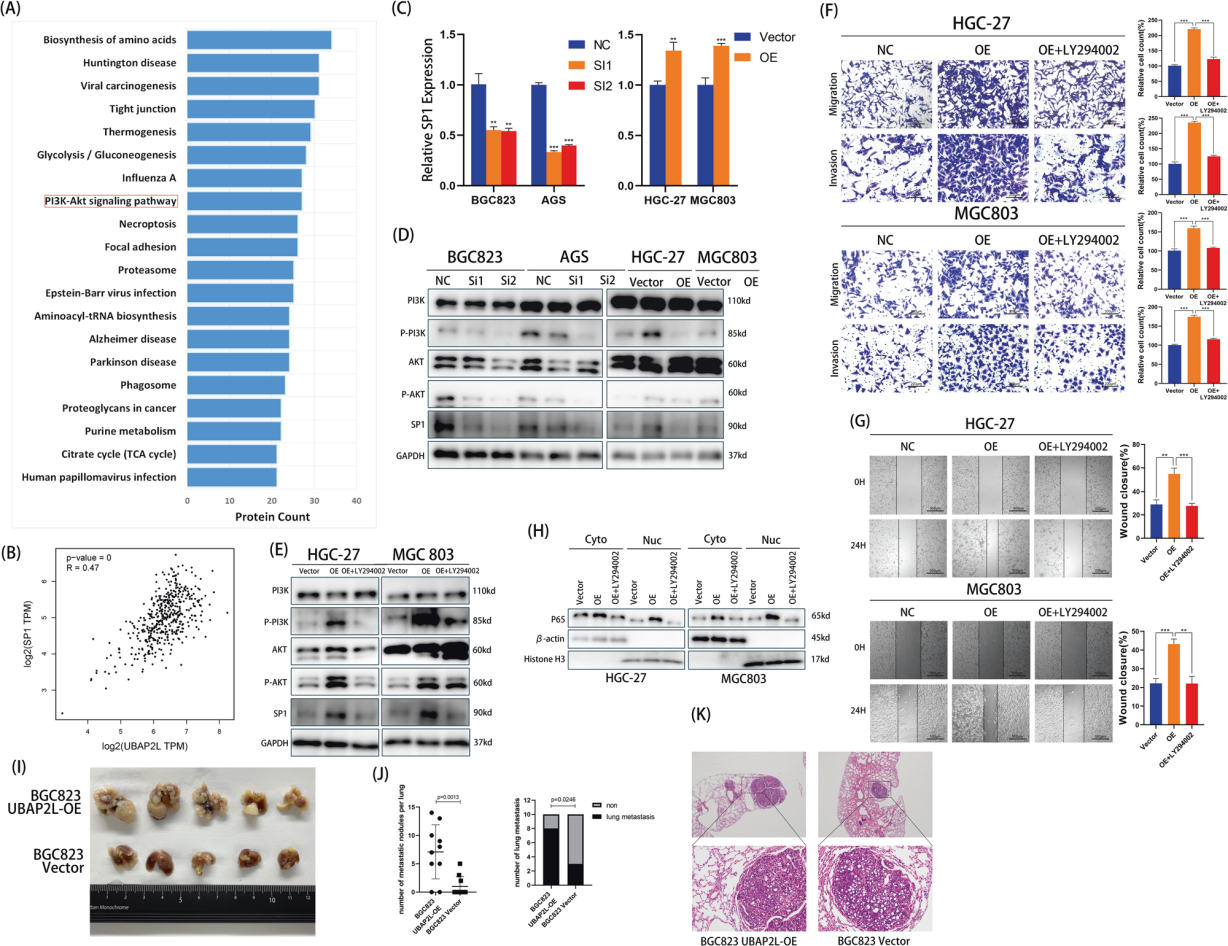
UBAP2L activates p65 through PI3K/AKT/SP1 axis. **A** Pathway annotation of 1244 proteins identified by mass spectrometry. **B** The relationship of mRNA between UBAP2L and SP1. **C** The mRNA level of SP1 was suppressed by the knockdown of UBAP2L in BGC823/AGS cells, and was increased in UBAP2L-overexpressed HGC-27/MGC803 cells. **D** Western blot analyzed the expression of PI3K, p-PI3K, AKT, p-AKT and SP1 in UBAP2L-silenced and UBAP2L-overexpressed cells. **E** The expression of PI3K, p-PI3K, AKT, p-AKT and SP1 were detected by western blot in UBAP2L-overexpression HGC-27/MGC803 cells treated with LY294002. **F**, **G** Transwell assay and wound healing assay were undertaken to measure the effects of LY294002 treatment on migration and invasion abilities of UBAP2L-overexpression HGC-27/MGC803 cells. **H** The nuclear translocation of p65 in UBAP2L-overexpression HGC27/MGC803 cells treated with LY294002. **I** Representative images of pulmonary metastatic nodules. **J** The number of metastatic nodules in each lung and the number of mice with pulmonary metastatic nodules in BGC823 UBAP2L-OE and BGC823 Vector groups. **K** Representative images of H&E staining of metastatic nodules in BGC823 UBAP2L-OE and BGC823 Vector groups.

Further, we examined how UBAP2L affects the activation of p65. Immunoprecipitation and mass spectrometry were used to identify proteins associated with UBAP2L. UBAP2L-binding proteins were identified by comparing the anti-FLAG IP products with those of the IgG control, and 1244 proteins were identified. Then, functional annotations including GO annotation, KOG annotation, and pathway annotation were performed. Surprisingly, pathway annotation showed that the PI3K/AKT pathway was involved (Fig. 6A). We then performed Co-IPs to demonstrate the association of UBAP2L with core proteins of the PI3K/AKT pathway in BGC823 and AGS cells (Supplementary Fig. 2). It has been reported that the PI3K/AKT pathway increases SP1 binding to the p65 promoter [20–23]. To assess whether UBAP2L activates p65 through the PI3K/AKT/SP1 axis, we first investigated the relationship between UBAP2L and SP1. As shown in Fig. 6B, the mRNA levels of UBAP2L showed a strong positive correlation with SP1 expression, determined using the TCGA database (*R* = 0.47, *p* < 0.001). Both mRNA and protein levels of SP1 were significantly suppressed when UBAP2L was knocked down in BGC823 and AGS cells. In contrast, the expression of SP1 was increased in UBAP2L-overexpressed HGC-27 and MGC803 cells (Fig. 6C, D). Furthermore, we tested the expression of PI3K and AKT as well as their phosphorylation levels. The results indicated that silencing of UBAP2L markedly downregulated the levels of p-PI3K and p-AKT; however, total PI3K and AKT levels showed no significant changes. In UBAP2L-overexpressed GC cells, total PI3K and AKT levels remained stable, and the levels of p-PI3K and p-AKT were markedly upregulated in comparison with that of the vector group (Fig. 6D).

Next, we performed rescue experiments in HGC-27 and MGC803 cell lines transfected with UBAP2L lentivirus with or without the PI3K/AKT pathway inhibitor LY294002. UBAP2L-overexpressed HGC-27 and MGC803 cells treated with LY294002 showed significant downregulation of p-PI3K and p-AKT levels (Fig. 6E). Transwell assays revealed that LY294002 dramatically suppressed the migration and invasion abilities of UBAP2L-overexpressed HGC-27 and MGC803 cells (Fig. 6F). The wound-healing assay exhibited a distinctly wider wound compared with that of the non-processing group (Fig. 6G). Further, we confirmed the hypothesis that LY294002 ultimately influences the movement of p65 from the cytoplasm to the nucleus. As shown in Fig. 6H, LY294002 attenuated the upregulation of nuclear protein P65 levels induced by UBAP2L overexpression. These results indicate that UBAP2L activates p65 via the PI3K/AKT/SP1 axis, and the blockage of the PI3K/AKT pathway reversed UBAP2L-induced nuclear translocation of p65.

### UBAP2L promotes the metastasis of GC in vivo

To investigate the biological function of UBAP2L in tumor metastasis in vivo, athymic nude mice were injected with BGC823 UBAP2L-OE and BGC823 Vector cells via the tail veins. The number and size of lung metastatic nodules were increased in the athymic nude mice injected with BGC823 UBAP2L-OE cells, while fewer and smaller nodules appeared in the BGC823 Vector group (Fig. 6I). In the BGC823 UBAP2L-OE group, the number of metastatic nodules per lung and the incidence of lung metastasis were considerably increased in comparison with that of the BGC823 Vector group (Fig. 6J). We also observed a higher degree of malignancy and larger metastatic lesions in the lung tissue (paraffin sections with HE staining) of the BGC823 UBAP2L-OE group (Fig. 6K). This suggests that UBAP2L plays an important role in tumor metastasis.

## Discussion

Although the incidence of GC has been declining in many parts of the world for decades, it is markedly elevated in eastern Asia [24]. The onset of GC is hidden, and there are no obvious symptoms during the early stage. Its 5-year survival ranges between 20% and 40% in most countries. The best potentially curative therapeutic approaches for GC are radical gastrectomy followed by adequate lymphadenectomy and chemotherapy [25]. Regrettably, only life-prolonging palliative care can be provided for patients with unresectable, locally advanced, or metastatic cancer [26]. Therefore, a deeper understanding of the molecular heterogeneity and wider screening of potential molecular targets are vital to boost treatments using personalized medicine [27]. Distant metastasis of tumors is regarded as the main cause of cancer-related deaths. However, its molecular mechanism is largely unknown [28].

Aberrant overexpression of UBAP2L has been shown to be involved in tumor growth and metastasis and predicts poor survival prognoses [8–10, 13–15]. However, its role in GC development and progression remains unclear. In the current study, we demonstrated that the expression of UBAP2L was upregulated in both GC tissues and cell lines. Higher expression of UBAP2L was also correlated with several poor clinicopathological characteristics, poor prognoses, and shorter survival times.

The expression of SP1 is stimulated through the PI3K/AKT pathway, and elevated levels of SP1 activate NF-κB transcription. Activation of NF-κB is ultimately associated with tumor aggression [29, 30]. The NF-κB transcription factor is a heterodimeric protein that was first identified based on its interaction with the immunoglobulin light-chain enhancer in B cells [31]. NF-κB is sequestered in the cytoplasm and is in a latent, inactive state and bound to an inhibitor. When the cells are exposed to several stimuli such as LPS or inflammatory cytokines, viral or bacterial infection, UV irradiation, and other physiological, chemical, or physical stimuli, NF-κB can be rapidly activated. The phosphorylation and subsequent degradation of IκBs allow these inactive complexes to dissociate, freeing NF-κB from their inhibitor and subsequently moving NF-κB into the nucleus. The translocation of NF-κB to the nucleus enables its dimers to bind to target DNA elements and then activate the transcription of genes [32–34]. The p50/p65 heterodimer is a prototypical NF-κB complex, and p65 phosphorylation plays a significant role in nuclear regulation of NF-κB transcriptional activity [35]. Therefore, in this study, we evaluated how the expression and nuclear translocation of p65 are influenced by UBAP2L. Correlation analysis of the mRNA levels suggested that there was a strong positive correlation between UBAP2L and p65. In addition, more p65 was translocated to the nucleus in UBAP2L over-expression cell lines, and a reduction in p65 nuclear translocation in cells with attenuated UBAP2L expression was observed through nuclear and cytoplasmic protein extraction and immunofluorescence experiments. We also conducted rescue experiments using Betulinic acid and BAY 11-7082. The results of Transwell assays, western blotting, and IF assays showed that, when Betulinic acid was added to knockdown UBAP2L cells, p65 nuclear translocation and the stronger metastasis ability were rescued. However, BAY 11-7082 treatment in UBAP2L-overexpression cell lines dramatically suppressed the upregulation of p65 nuclear protein levels and metastasis phenotypes. These results clearly demonstrate that UBAP2L is was a positive regulator of NF-κB, and p65 is positively associated with the mechanism involved in UBAP2L-mediated regulation of GC metastasis.

After establishing the type of relationship between UBAP2L and p65, we explored the mechanism of UBAP2L-mediated elevation of p65 expression and activation. We conducted mass spectrometry to explore the downstream pathways of UBAP2L and the PI3K/AKT pathway was in the results. The PI3K/AKT pathway plays a central role in the regulation of multiple cellular processes, including sustaining proliferative signaling, evading growth suppressors, resisting cell death, enabling replicative immortality, inducing angiogenesis, and activating invasion and metastasis [36, 37]. The phosphorylation of two key residues of AKT1, T308, and S473, is primarily regulated by the activation of PI3K [38]. AKT directly phosphorylates substrate proteins that are included in numerous functional classes, including protein kinases, transcription factors, metabolic enzymes, E3 ubiquitin ligases, and cell cycle regulators [39]. Activation of the PI3K/AKT pathway has long been known as an important link in promoting the progression of GC [40].

The expression of p65 is stimulated by SP1. As described previously, the p65 promoter contains three potential SP1-binding sites (GGCGGG) [41], and SP1 increases the promoter activity of p65 by binding to the p65 promoter [20]. Previous studies have demonstrated that AKT transactivating SP1, mediates its binding to the target gene promoter [23, 42, 43]. To investigate whether SP1 is regulated by UBAP2L through the PI3K/AKT pathway, we analyzed the relationship between UBAP2L and SP1. We observed that the expression of SP1 was increased by UBAP2L at both the mRNA and protein levels. We also verified that the upregulated expression of SP1 was induced by the PI3K/AKT pathway. We observed that silencing UBAP2L markedly downregulated the levels of p-PI3K, p-AKT, and SP1. In contrast, the levels of p-PI3K, p-AKT, and SP1 were significantly elevated in UBAP2L-overexpressed GC cells. Treatment with LY294002 partially blocked the UBAP2L-induced activation of the PI3K/AKT pathway and the upregulation of SP1 expression. From these results, we established that UBAP2L activates NF-κB through the PI3K/AKT/SP1 axis.

## Conclusion

**Fig. 7:**
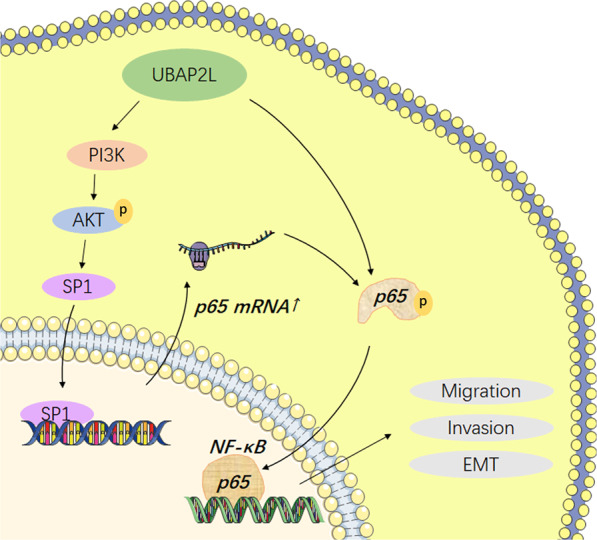
The regulatory network of UBAP2L in gastric cancer. Aberrant expression of UBAP2L activates p65 through PI3K/AKT/SP1 axis. The increase of p65 nuclear translocation promotes the migration and invasion abilities of GC cells and influences the expression of EMT markers.

In summary, we observed that UBAP2L expression was upregulated in GC tissues and cell lines. Our study provides new insights into the critical role of UBAP2L in facilitating GC metastasis. Western blotting, immunofluorescence, mass spectrometry, and rescue assay demonstrated the involvement of NF-κB in the UBAP2L-induced EMT process in GC cells and elucidated that the activation of NF-κB is dependent on the activation of the PI3K/AKT/ SP1 axis (Fig. 7). Therefore, targeting UBAP2L may be a potential strategy for GC therapy.

## Materials and methods

### Tissue specimens and clinicopathological data

GC tissues and matched adjacent normal mucosae were obtained from the Department of General Surgery at Xinhua Hospital affiliated to Shanghai Jiao Tong University School of Medicine (Shanghai, China). None of the patients were treated with radiotherapy or chemotherapy before undergoing surgical resection. Approval and written informed consent were obtained from the Research Ethics Committee of Xinhua Hospital and all the patients, respectively, before commencing all the experiments. The cancer stage of each patient was assessed according to the 8th edition of the American Joint Committee on Cancer Staging Manual.

### Cell culture and reagents

Four human GC cell lines (HGC-27, MGC803, BGC823, and AGS) and one normal gastric mucosal epithelial cell line (GES-1) were used in the present study. All cells were purchased from the Cell Bank of the Chinese Academy of Sciences (Shanghai, China). The cells were maintained in RPMI-1640 medium (Hyclone) supplemented with 10% fetal bovine serum (FBS) (Invitrogen Gibco) at 37 °C in a humidified chamber containing 5% CO_2_. Cells in the logarithmic growth phase were used for subsequent assays. LY294002 was purchased from MedChemExpress, and Betulinic acid and BAY 11-7082 were generously provided by Haojie Chen (Xinhua hospital). All reagents were dissolved in DMSO. Cells were treated with these reagents at the recommended concentration (Betulinic acid: 50 μM, BAY 11-7082: 15 μM, LY294002: 20 μM) for a certain period before conducting the subsequent experiments.

### Cell transfection

To knock down the expression of UBAP2L, three small interfering RNAs (siRNAs) targeting UBAP2L and the parental negative control (NC) were synthesized by Genomeditech (Shanghai, China). RFect reagent (Baidai, China) was used for transfection according to the manufacturer’s protocol. Ectopic UBAP2L overexpression lentivirus was constructed according to the human UBAP2L full-length sequence by Genomeditech (Shanghai, China), and an empty vector was used as the control. Lentivirus infection was performed according to the manufacturer’s instructions. Western blotting and quantitative real-time PCR were used to validate transfection efficiency. The sequences of siRNA and full-length UBAP2L are provided in the Supplementary Materials.

### Quantitative real-time PCR (qRT-PCR)

Total RNA was extracted from cells using the Trizol reagent (Invitrogen). cDNA was generated using the PrimeScript RT reagent kit with gDNA Eraser (TaKaRa) according to the manufacturer’s instructions. qRT-PCR was conducted using SYBR premix Ex Taq (TaKaRa) according to the manufacturer’s instructions. A StepOnePlus^TM^ Real-time PCR system (Applied Biosystems, USA) was used.

### Cell invasion and migration assays

Transwell chamber inserts (Corning, NY, USA) and Corning BioCoat Growth Factor Reduced Matrigel Invasion Chambers (Corning, NY, USA) were used for the migration and invasion assays. HGC-27 (2 × 10^4^ cells per well), MGC803 (3 × 10^4^ cells per well), BGC823 (8 × 10^4^ cells per well), and AGS (10 × 10^4^ cells per well) were resuspended in 200 μL of medium without FBS and then placed in the upper chamber. The substrates were placed in a 24-well plate filled with 600 μL of complete medium. The plates were incubated in a humidified incubator at 37 °C containing 5% CO_2_ for 24 h. After incubation, the cells were fixed in 4% paraformaldehyde for 30 min and then stained with crystal violet for 30 min. Five random fields were selected under an inverted microscope at ×100 magnification, where images were taken and the cell number was counted.

### Wound-healing assay

GC cells were seeded and reached at least 90% confluence in a 6-well plate. The monolayer of cells was scratched in a straight line to generate wounds with a uniform breadth using a 200 μL pipette tip and then rinsed with sterile PBS three times. Next, 2 mL medium without FBS was added to each well, and the plate was incubated for 24 h before calculating the wound closure percentage. The width of the wounds at 0 and 24 h was recorded and measured at five random fields.

### Western blot analysis

Protein extraction and western blotting were performed as previously described [44]. In brief, proteins were isolated with RIPA Lysis buffer (Beyotime) and quantified using BCA assay (Beyotime). Proteins were separated by SDS-PAGE and transferred onto PVDF membranes (Millipore). The membranes were blocked with 5% skimmed milk for 1 h and then incubated with primary antibodies at 4 °C overnight. The next day, the membranes were incubated with the secondary antibodies (Beyotime). The immunoreactive bands were detected by chemiluminescence and visualized using a Gel Doc 2000 (Bio-Rad, USA). The antibodies used are presented in the Supplementary Materials.

### Immunoprecipitation and mass spectrometry

Cells were lysed using IP Lysis Buffer (Beyotime). The supernatant was collected after centrifugation and then incubated with anti-FLAG primary antibody or IgG at 4 °C overnight. Protein A + G agarose (Beyotime) was added to the supernatant the next day and the mixture was rotated for 4 h at 4 °C. Finally, the Protein A + G agarose was washed five times with PBS, followed by further analysis.

The sample proteins were separated using gel electrophoresis, and protein gel strips were obtained at different positions on the film. Trypsin enzyme was added to 100 μg of protein according to the trypsin enzyme (μg): substrate protein (μg) ratio of 1:20. The mixture was vortexed, centrifuged at low speed for 1 minute, and incubated at 37 °C for 4 h. After desalting, the peptide was freeze-dried. The dried peptide samples were reconstituted with mobile phase A (2% ACN, 0.1% FA), and separated using Thermo UltiMate 3000 UHPLC. The peptides separated by liquid-phase chromatography were ionized by a nanoESI source and then passed to a tandem mass spectrometer Q-Exactive HF X (Thermo Fisher Scientific, San Jose, CA) for DDA (Data Dependent Acquisition) mode detection.

### Subcellular fractionation

Nuclear and cytoplasmic fractions were isolated using Nuclear and Cytoplasmic Extraction Reagents (78835, Thermo Fisher Scientific), according to the manufacturer’s instructions. The subcellular distribution of proteins was determined using western blot analysis. β-actin and Histone H3 served as loading controls for cytosolic and nuclear fractions, respectively.

### Immunohistochemistry

Tissues from 150 GC patients were used for immunohistochemistry. The tissues were embedded in paraffin initially. The paraffin-embedded tissue sections were then cut, and the sections were mounted on slides. Immunohistochemistry was performed according to a standard protocol. The IHC score was calculated according to the staining extent and intensity as follows: negative (1): <10% immunoreactive cells; weak (2): 10–49% immunoreactive cells; moderate (3): 50–74% immunoreactive cells; strong (4): ≥75% immunoreactive cells. The staining color was scored as no staining (0), light yellow (1), brownish yellow (2), and brown (3). The final score was calculated by multiplying the two scores. Scores below 5 were defined as low expression, and scores exceeding 5 were defined as high expression. Based on the above criteria, 107 GC tissues were classified under the UBAP2L-high group, and 43 tissues were classified under the UBAP2L-low group.

### Immunofluorescence assay

GC cells were seeded on coverslips one day before and fixed using 4% paraformaldehyde for 30 min at room temperature. The cells were incubated for 30 min with 0.5% Triton X-100 in PBS, followed by incubation with 5% BSA for 30 min to block non-specific binding sites of the antibodies. Then, the cells were incubated with specific primary antibodies diluted according to the manufacturer’s instructions in a humidified chamber at 4 °C overnight. The next day, the cells were incubated with the fluorescent secondary antibody for 1 h at room temperature in the dark. Finally, DAPI was used to stain the nuclei for 5 min. A fluorescence microscope (Leica DM4B) was used to observe the fluorescence intensity and capture images.

### Cell viability assay

Cell viability was evaluated by the CCK-8 assay and colony-forming assay. For the CCK-8 assay, cells were seeded in a 96-well plate at a density of 1000 cells per well. On days 1–5, the complete medium was discarded and 100 μL of the fresh medium containing 10% CCK-8 reagent (Yeasen, Shanghai, China) was added to each well, followed by incubation for 2 h at 37 °C in the dark. Then, the OD value at 450 nm was measured using a SpectraMax 190 Microplate Reader. For the colony-forming assay, cells were seeded in a 6-well plate at a density of 500 cells per well for 2 weeks. Each well was fixed with paraformaldehyde for 15 min before staining with crystal violet for 15 min. Colonies with >50 cells were counted, and the images were taken.

### Flow cytometry

Flow cytometry was used to perform the apoptosis and cell cycle assays. Annexin V-FITC kit (BD Biosciences, CatLog: 556547) and Cell Cycle Analysis Kit (Beyotime) were used to analyze the apoptosis and cell cycle of GC cells, respectively, according to the manufacturer’s protocol. Afterward, the samples were analyzed using flow cytometry (CytoFLEX, Beckman Coulter).

### Pulmonary metastasis model in nude mice

Nude mice (4 weeks old) were acquired from the Shanghai Laboratory Animal Center of the Chinese Academy of Sciences (Shanghai, China) and housed under pathogen-free conditions. 20 mice were divided into two groups randomly. Each mouse was injected with 5 × 10^6^ BGC823 UBAP2L-OE or BGC823 Vector cells through the lateral tail vein. 4 weeks after the initial implantation, all mice were sacrificed by cervical dislocation, and the lung tissues were isolated for further study. Animal feeding and experiments were carried out in strict accordance with the National Institutes of Health Guide for the Care and Use of Laboratory Animals (NIH Publications No. 8023, revised 1978). All animal studies were approved by the Ethics Committee of Xinhua Hospital affiliated to Shanghai Jiao Tong University School of Medicine.

### Statistical analysis

Statistical analyses were performed primarily on Prism 8 (GraphPad Software) and SPSS 24 (SPSS Inc.). All experiments were repeated at least three times independently, and the data are presented as the mean ± standard deviation (SD). Student’s *t* test was performed for the comparisons of quantitative variables between two groups, and analysis between multiple groups was conducted using one-way analysis of variance. Associations between UBAP2L expression and clinicopathological parameters were calculated using Pearson’s *χ*^2^ test. Kaplan–Meier test was used for univariate survival analysis. Fisher’s exact test was applied for the analyses of different incidences of lung metastasis. *p* Value <0.05 was considered statistically significant (*p < 0.05, **p < 0.01, ***p < 0.001).

## Supplementary information

original western blots

Supplementary Materials

## Data Availability

The data generated or analyzed during this study are included in this published article. Please contact authors for other data requests. The datasets used and analyzed during the current study are available from the corresponding authors on reasonable request.
