# Supplementary material for: UBAP2L promotes gastric cancer metastasis by activating NF-κB through PI3K/AKT pathway
Source: Cell Death Discov. 2022 Mar 19;8:123. doi: 10.1038/s41420-022-00916-7 (PMC8933503; doi:10.1038/s41420-022-00916-7)

**Figure 1**

UBAP2L


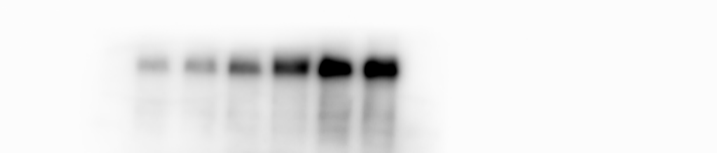


GAPDH


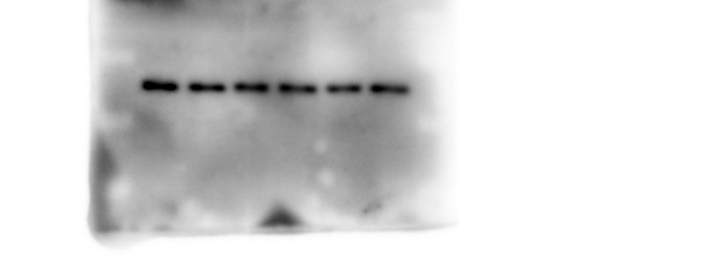


**Figure 2**

UBAP2L


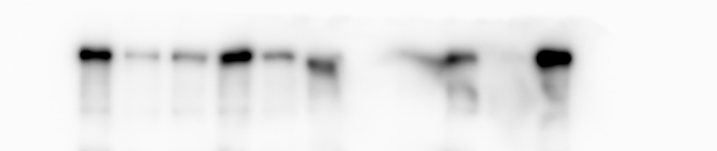


GAPDH


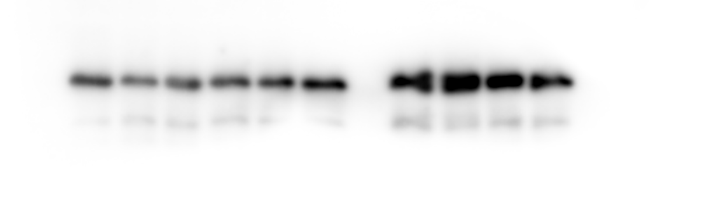


**Figure 3**

UBAP2L


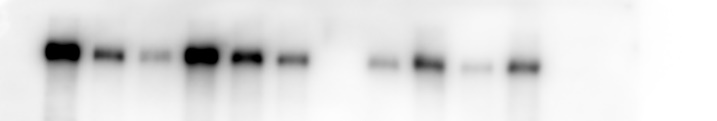


E-cadherin


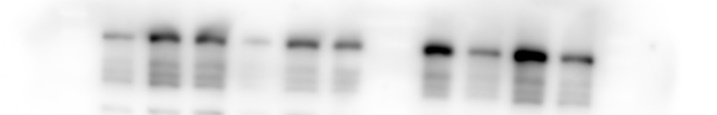


N-cadherin


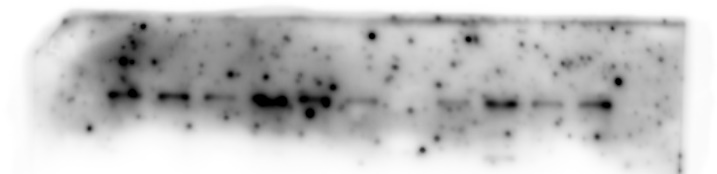


Vimentin


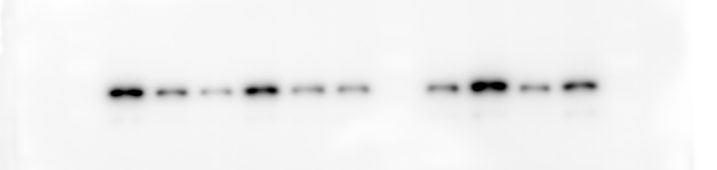


β-catenin


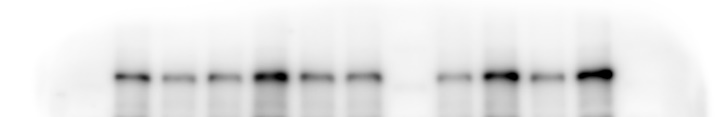


GAPDH


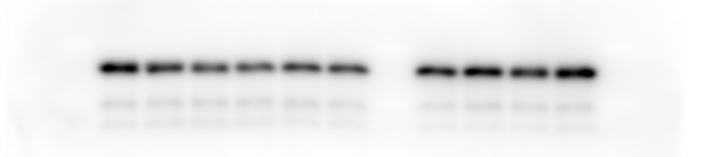


**Figure 4c**

P65


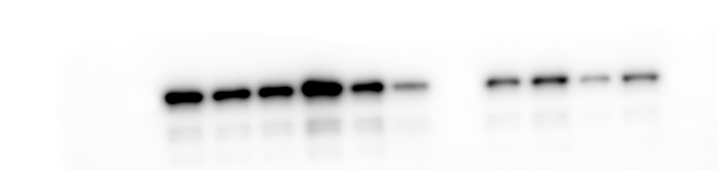


P-P65


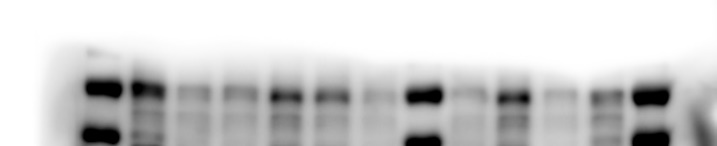


GAPDH


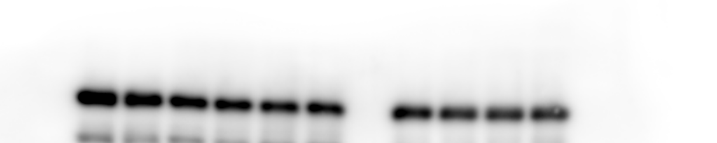


**Figure 4D**

P65


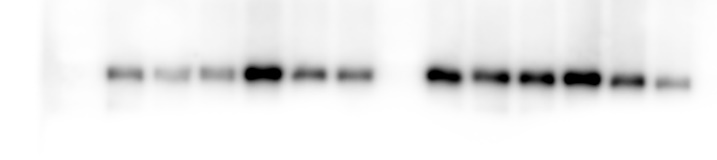

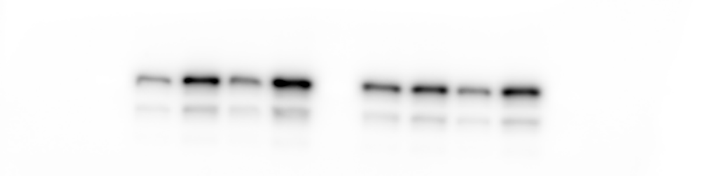


β-actin


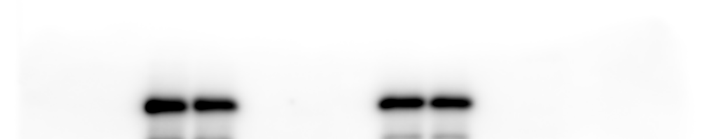

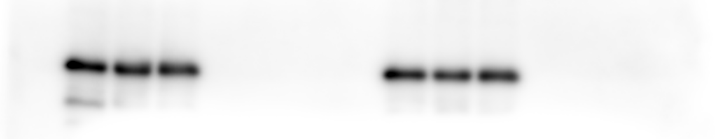


Histon H3


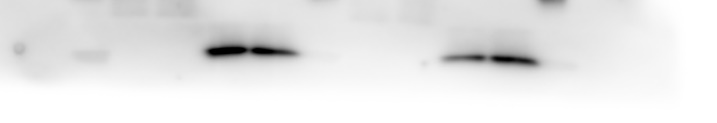

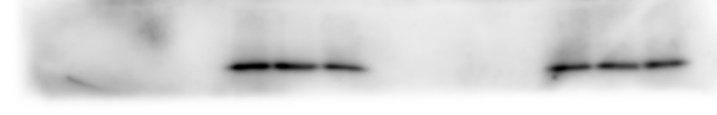


**Figure 5E**

P65


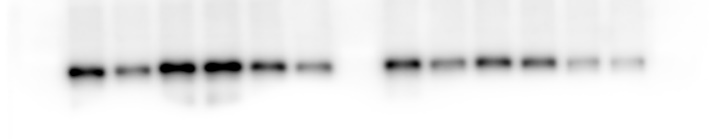

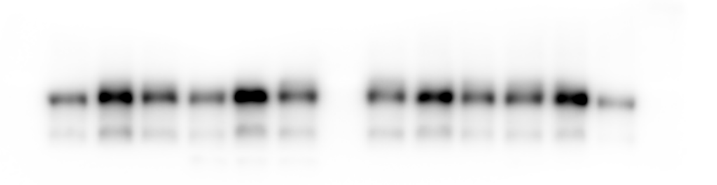


β-actin


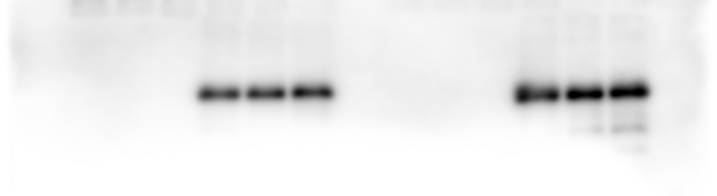

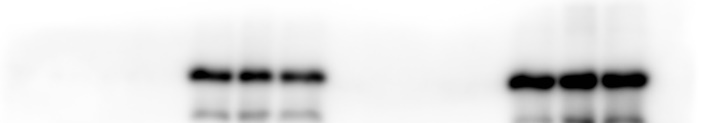


Histone H3


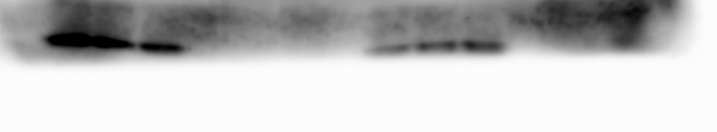

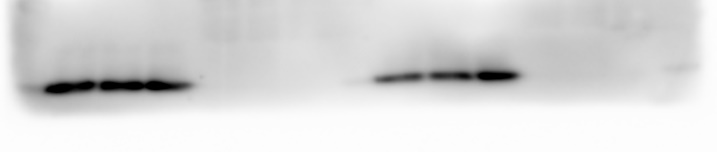


**Figure 5F**

E-cadherin


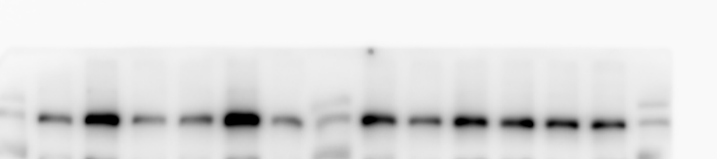


N-cadherin


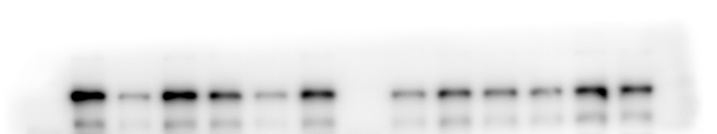


Vimentin


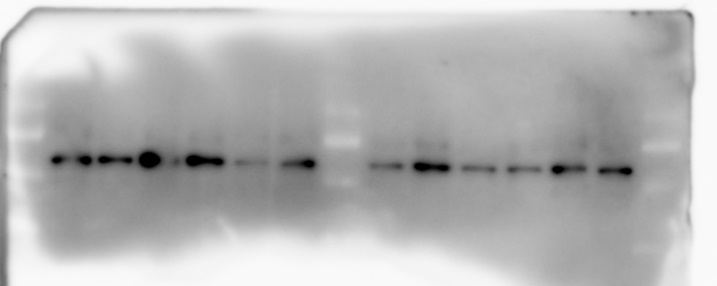


β-catenin


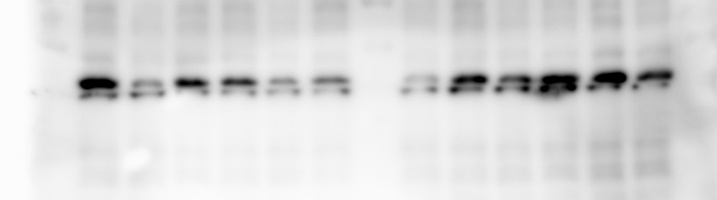


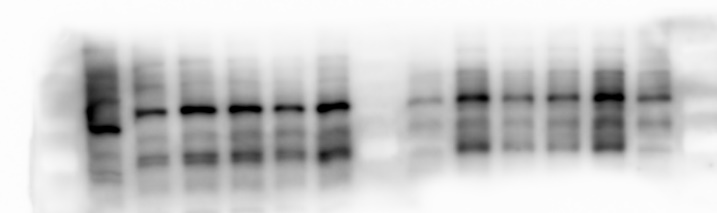


GAPDH


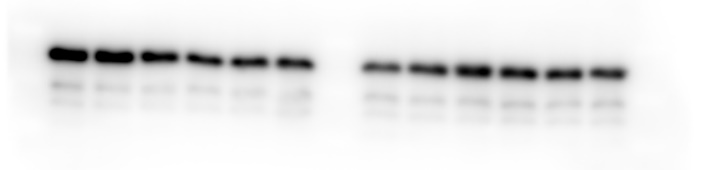


**Figure 6D**

PI3K, AKT


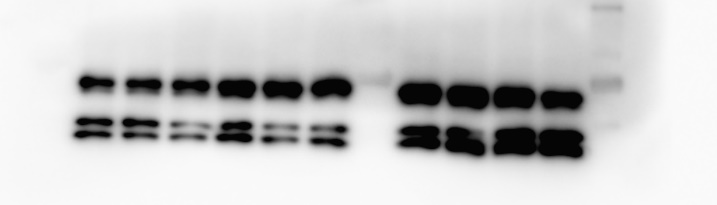


P-PI3K


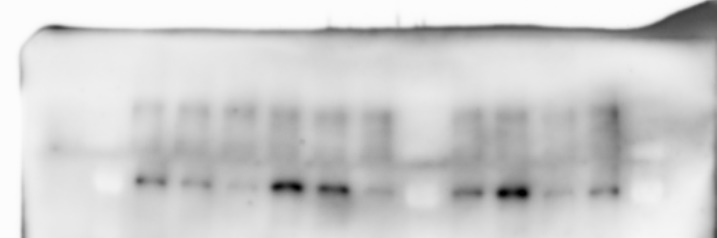


P-AKT


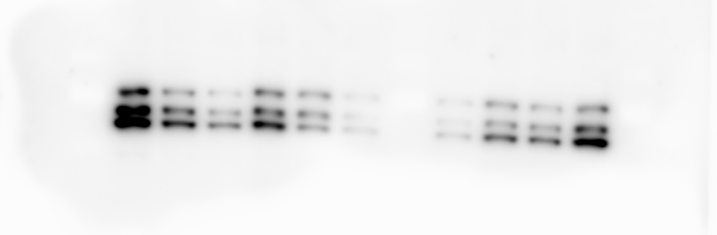


SP1


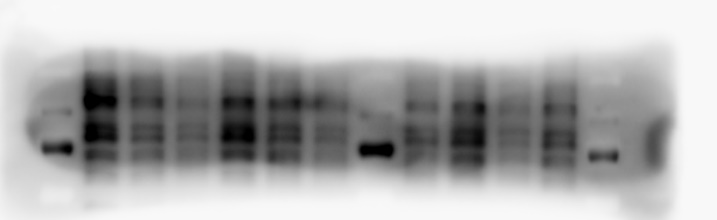


GAPDH


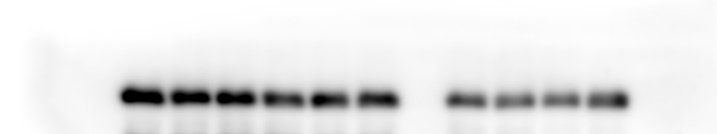


**Figure 6E**

PI3K, AKT


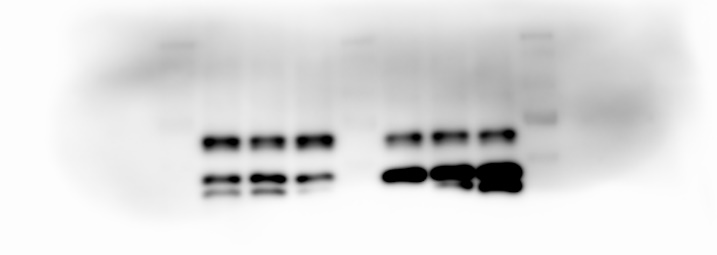


P-PI3K


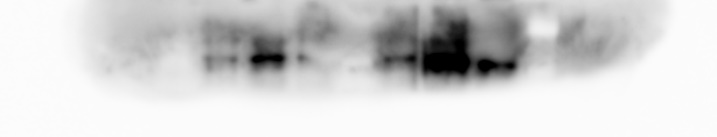


P-AKT


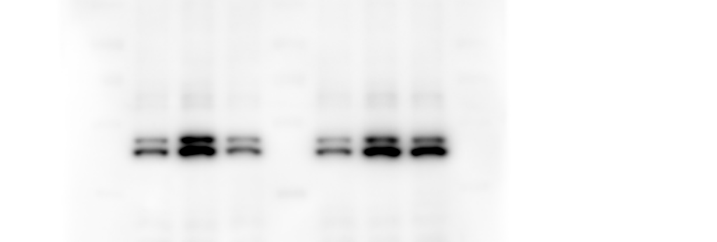


SP1


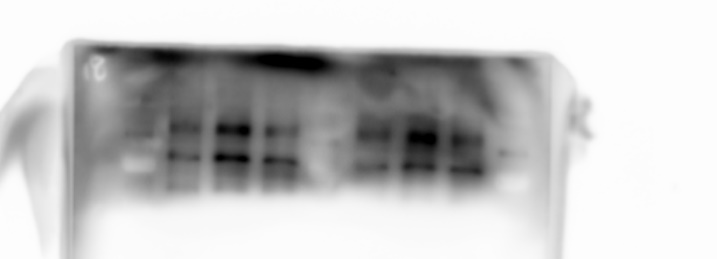


GAPDH


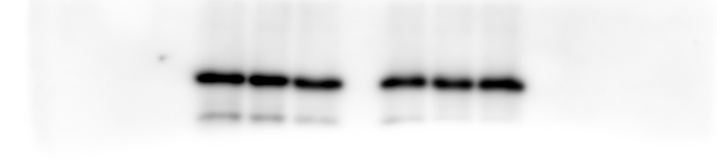


**Figure 6H**

P65


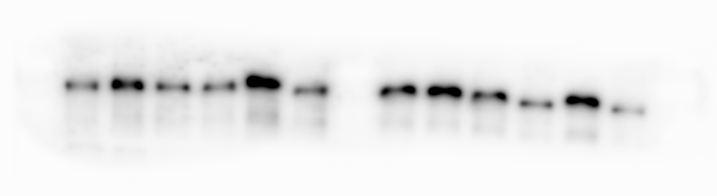


β-actin


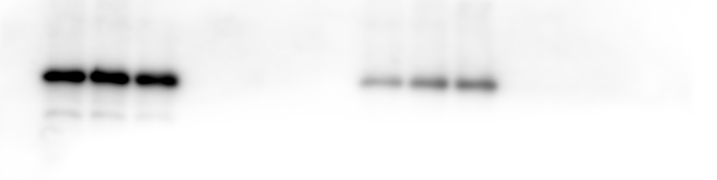


Histone H3


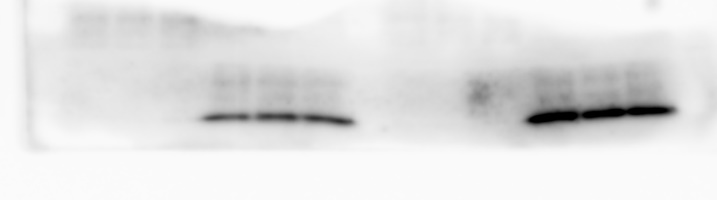


**Supplement figure 2**

UBAP2L


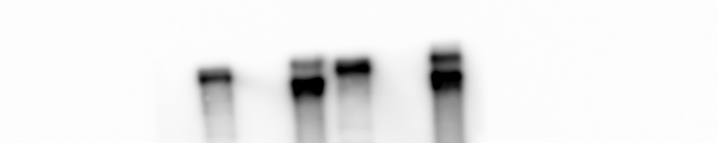


Hsp90β


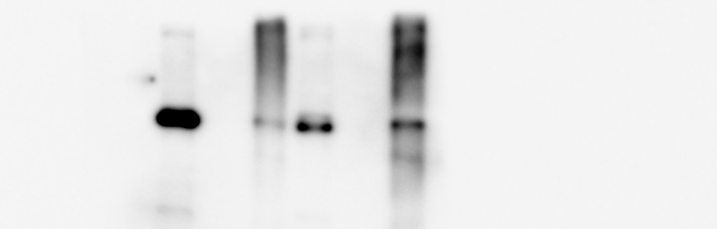


CDC37


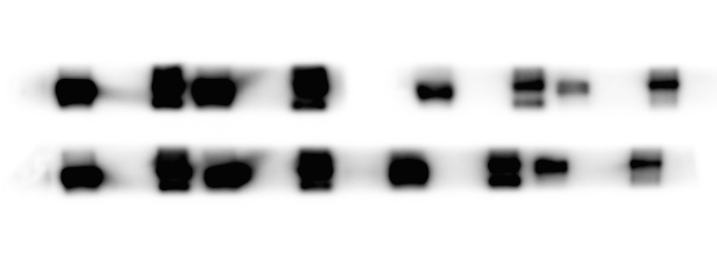

Supplement: Supplementary file 1 — original western blots [file 41420_2022_916_MOESM1_ESM.docx]
