# Supplementary material for: UBAP2L promotes gastric cancer metastasis by activating NF-κB through PI3K/AKT pathway
Source: Cell Death Discov. 2022 Mar 19;8:123. doi: 10.1038/s41420-022-00916-7 (PMC8933503; doi:10.1038/s41420-022-00916-7)
Supplement: Supplementary file 2 — Supplementary Materials [file 41420_2022_916_MOESM2_ESM.docx]

**Supplementary Materials and Methods**

**Antibodies used**

Antibodies used in western blot: UBAP2L(ab70319, Abcam, 1:1000), PI3K(#4249, Cell Signaling Technology, 1:1000) , p-PI3K(#17366, Cell Signaling Technology, 1:1000), AKT(A17909, ABclonal, 1:1000), p-AKT(#4060, Cell Signaling Technology, 1:1000), E-cadherin(#3195, Cell Signaling Technology, 1:1000), N-cadherin(#13116, Cell Signaling Technology, 1:1000), Vimentin(#5741, Cell Signaling Technology, 1:1000), β-catenin(#8480, Cell Signaling Technology, 1:1000), SP1(A19649, ABclonal, 1:1000), GAPDH(#5174, Cell Signaling Technology, 1:1000), Histon H3(A17562, ABclonal, 1:1000), β-actin(#4970, Cell Signaling Technology, 1:1000), NF-κB p65(#8242, Cell Signaling Technology, 1:1000), p-NF-κB p65(#3033 Cell Signaling Technology, 1:1000), Hsp90β(A1087, ABclonal, 1:1000), CDC37(A4582, ABclonal, 1:1000), secondary antibody(A0208, Beyotime, 1:1000).

Antibodies used in IF assays: NF-κB p65(#8242 Cell Signaling Technology, 1:400).

Antibodies used in Co-IP: FLAG(66008-3-Ig Proteintech), IgG(A7028 Beyotime).

**The primers used**

| GAPDH | forward | 5’-CAACAGCCTCAAGATCATCAGC-3’ |
| --- | --- | --- |
|  | reverse | 5’-TTCTAGACGGCAGGTCAGGTC-3’ |
| UBAP2L | forward | 5’- AGCCGTGGACGAGAGTTTC-3’ |
|  | reverse | 5’-CGTATTGCCGCTGCTATTGC-3’ |
| RELA(p65) | forward | 5’-AGGCGAGAGGAGCACAGATACCAC-3’ |
|  | reverse | 5’-TCCCGGCAGTCCTTTCCTACAAGC-3’ |
| SP1 | forward | 5’- ATGTTGCCTCCACTTCCTCGATTTG-3’ |
|  | reverse | 5’-GCACAGTCTCTGGTGGGCAGTATGT-3’ |

**The siRNA used**

| UBAP2L（h）-si-1 | GCCAAUACUGAUGAUAACU tt |
| --- | --- |
| UBAP2L（h）-si-2 | GGUGAUGUCGGUGAAGCUA tt |
| UBAP2L（h）-si-3 | GGGAAGACACCAUCUACAA tt |

**The UBAP2L sequence used for overexpression:**

| **plasmid** | **PGMLV-CMV-H_UBAP2L-3×Flag-PGK-Puro** |
| --- | --- |
| CATATGCCAAGTACGCCCCCTATTGACGTCAATGACGGTAAATGGCCCGCCTGGCATTATGCCCAGTACATGACCTTATGGGACTTTCCTACTTGGCAGTACATCTACGTATTAGTCATCGCTATTACCATGGTGATGCGGTTTTGGCAGTACATCAATGGGCGTGGATAGCGGTTTGACTCACGGGGATTTCCAAGTCTCCACCCCATTGACGTCAATGGGAGTTTGTTTTGGCACCAAAATCAACGGGACTTTCCAAAATGTCGTAACAACTCCGCCCCATTGACGCAAATGGGCGGTAGGCGTGTACGGTGGGAGGTCTATATAAGCAGAGCTCTCTGGCTAACTAGAGAACCCACTGCTTACTGGCTTATCGAAATTAATACGACTCACTATAGGGAGACCCAAGCTGGCTAGTTAAGCTTGGTACCGAGCTCGGATCCGCCACCATGATGACATCGGTGGGCACTAACCGAGCCCGGGGAAACTGGGAACAACCTCAAAACCAAAACCAGACACAGCACAAGCAGCGGCCACAGGCCACTGCAGAACAAATTAGACTTGCACAGATGATTTCGGACCATAATGATGCTGACTTTGAGGAGAAGGTGAAACAATTGATTGATATTACAGGCAAGAACCAGGATGAATGTGTGATTGCTTTGCATGACTGCAATGGAGATGTCAACAGAGCTATCAATGTTCTTCTGGAAGGAAACCCAGACACGCATTCCTGGGAGATGGTCGGGAAGAAGAAGGGAGTCTCAGGCCAGAAGGATGGTGGCCAGACGGAATCCAATGAGGAAGGCAAAGAAAATCGAGACCGGGACAGAGACTATAGTCGGCGACGTGGTGGGCCACCAAGACGGGGGAGAGGTGCCAGCCGTGGACGAGAGTTTCGAGGTCAGGAAAATGGATTGGATGGCACCAAGAGTGGAGGGCCTTCTGGAAGAGGAACAGAAAGAGGCAGAAGGGGCCGTGGCCGAGGCAGAGGTGGCTCTGGTAGGCGAGGAGGAAGGTTTTCTGCTCAAGGAATGGGAACCTTTAACCCAGCTGATTATGCAGAGCCAGCCAATACTGATGATAACTATGGCAATAGCAGCGGCAATACGTGGAACAACACTGGCCACTTTGAACCAGATGATGGGACGAGTGCATGGAGGACTGCAACAGAGGAGTGGGGGACTGAAGATTGGAATGAAGATCTTTCTGAGACCAAGATCTTCACTGCCTCTAATGTGTCTTCAGTGCCTCTGCCTGCGGAGAATGTGACAATCACTGCTGGTCAGAGAATTGACCTTGCTGTTCTGCTGGGGAAGACACCATCTACAATGGAGAATGATTCATCTAATCTGGATCCGTCTCAGGCTCCTTCTCTGGCCCAGCCTCTGGTGTTCAGTAATTCGAAGCAGACTGCCATATCACAGCCTGCTTCAGGGAACACATTTTCTCATCACAGTATGGTGAGCATGTTAGGGAAAGGATTTGGTGATGTCGGTGAAGCTAAAGGCGGCAGTACTACAGGCTCCCAGTTCTTGGAGCAATTCAAGACTGCCCAAGCCCTGGCTCAGTTGGCAGCTCAGCATTCTCAGTCTGGAAGCACCACCACCTCCTCTTGGGACATGGGCTCGACGACACAATCCCCATCACTGGTGCAGTATGATTTGAAGAACCCAAGTGATTCAGCAGTGCACAGCCCCTTTACAAAGCGCCAGGCTTTTACCCCATCTTCAACCATGATGGAGGTGTTCCTTCAGGAGAAGTCACCTGCAGTGGCTACCTCCACAGCTGCACCTCCACCTCCGTCTTCTCCTCTGCCAAGCAAATCCACATCGGCTCCACAGATGTCGCCTGGATCTTCAGACAACCAGTCCTCTAGCCCTCAGCCGGCTCAGCAGAAACTGAAACAGCAGAAGAAAAAAGCCTCCTTGACTTCTAAGATTCCTGCTCTGGCTGTGGAGATGCCTGGCTCAGCAGATATCTCAGGGCTAAACCTGCAGTTTGGGGCATTGCAGTTTGGGTCAGAGCCTGTCCTTTCTGATTATGAGTCCACCCCCACCACGAGCGCCTCTTCAAGCCAGGCTCCAAGTAGCCTGTATACCAGCACGGCCAGTGAATCATCCTCTACAATTTCATCTAACCAGAGTCAGGAGTCTGGTTATCAGAGCGGCCCAATTCAGTCGACAACCTATACCTCCCAAAATAATGCTCAGGGCCCTCTTTATGAACAGAGATCCACACAGACTCGGCGGTACCCCAGCTCCATCTCTTCATCACCCCAAAAGGACCTGACTCAGGCAAAGAATGGCTTCAGTTCTGTGCAGGCCACGCAGTTACAGACCACACAATCTGTTGAAGGTGCTACAGGCTCTGCAGTGAAATCTGATTCACCTTCCACTTCTAGCATCCCCCCTCTCAATGAAACGGTATCTGCAGCTTCCTTACTGACGACAACCAATCAGCATTCATCCTCCTTGGGTGGCTTGAGCCACAGTGAGGAGATTCCAAATACTACCACCACACAACACAGCAGCACGTTATCTACGCAGCAGAATACCCTTTCATCATCAACATCTTCTGGGCGCACTTCGACATCCACTCTTTTGCACACAAGTGTGGAGAGTGAGGCGAATCTCCATTCTTCCTCCAGCACTTTTTCCACCACATCCAGCACAGTCTCTGCACCTCCCCCAGTGGTCAGTGTCTCCTCCAGTCTCAATAGTGGCAGTAGCCTGGGCCTCAGCCTAGGCAGCAACTCCACTGTCACAGCCTCGACTCGAAGCTCAGTTGCTACGACTTCAGGAAAAGCTCCTCCCAACCTCCCTCCTGGGGTCCCGCCGTTGTTGCCTAATCCGTATATTATGGCTCCAGGGCTGTTACATGCCTACCCGCCACAAGTATATGGTTATGATGACTTGCAGATGCTTCAGACAAGATTTCCATTGGATTACTACAGCATCCCATTTCCCACACCCACTACTCCGCTGACTGGGAGGGATGGTAGCCTGGCCAGCAACCCTTATTCTGGTGACCTCACAAAGTTCGGCCGTGGGGATGCCTCCTCCCCAGCCCCGGCCACAACCTTGGCCCAACCCCAACAGAACCAGACGCAGACTCACCATACCACGCAGCAGACATTCCTGAACCCGGCGCTGCCTCCTGGCTACAGTTACACCAGCCTGCCATACTATACAGGGGTCCCGGGCCTCCCCAGCACCTTCCAGTATGGGCCTGCTGTGTTCCCTGTGGCTCCTACCTCTTCCAAGCAGCATGGTGTGAATGTCAGTGTGAATGCATCGGCCACCCCTTTCCAACAGCCGAGTGGATATGGGTCTCATGGATACAACACTGGTGTTTCAGTCACCTCCAGTAACACGGGCGTGCCAGATATCTCGGGTTCTGTGTACTCCAAAACCCAGCAGTCCTTTGAGAAACAAGGTTTTCATTCCGGTACTCCTGCTGCTTCCTTCAACTTGCCTTCAGCCCTAGGAAGTGGGGGCCCCATCAATCCGGCCACAGCTGCTGCCTACCCACCTGCCCCCTTTATGCACATTCTGACCCCCCATCAGCAGCCGCATTCTCAGATCCTTCACCATCACCTGCAGCAGGATGGCCAGACGGGCAGCGGGCAACGTAGCCAGACCAGCTCCATCCCGCAGAAGCCCCAGACCAACAAGTCTGCCTACAACAGCTACAGCTGGGGGGCCAACCTCGAG | |
| 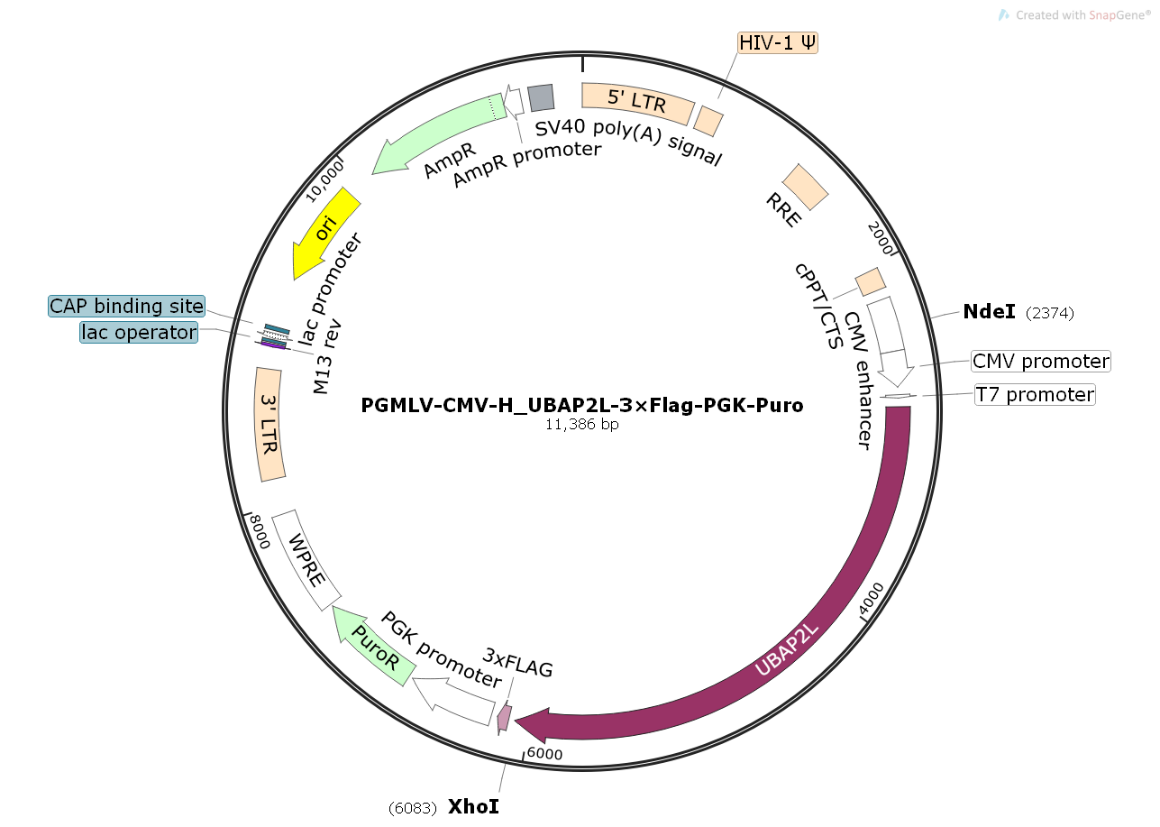 | |

**Supplement table 1: the expression of genes at chromosome 1q21.3**

| Name | Gene expression | | Name | Gene expression | |
| --- | --- | --- | --- | --- | --- |
|  | Tumor | Normal |  | Tumor | Normal |
| CTSK | 39.2 | 16.67 | ARNT | 16.7 | 11.4 |
| MCL1 | 114.44 | 83.99 | ADAR | 83.03 | 32.51 |
| NPR1 | 1.5 | 3.95 | SELENBP1 | 36.07 | 117.05 |
| PBXIP1 | 32.46 | 46.14 | SLC27A3 | 14.28 | 36.21 |
| PI4KB | 30.21 | 30.34 | CRTC2 | 27.24 | 24.31 |
| S100A13 | 50.37 | 49.83 | PMVK | 27.15 | 24.42 |

Red: upregulation. Green: downregulation. Gray: indiscrimination.

**Supplement table 2: Proteins in** **PI3K-Akt signaling pathway**

| **Pathway** | **Proteins** |
| --- | --- |
| PI3K-Akt signaling pathway | sp\|P01861\|IGHG4_HUMAN,sp\|P06730\|IF4E_HUMAN,sp\|P07900\|HS90A_HUMAN,sp\|P08238\|HS90B_HUMAN,sp\|P23588\|IF4B_HUMAN,sp\|P27348\|1433T_HUMAN,sp\|P28482\|MK01_HUMAN,sp\|P62258\|1433E_HUMAN,sp\|Q13131\|AAPK1_HUMAN,sp\|Q16543\|CDC37_HUMAN,tr\|A0A0S2Z430\|A0A0S2Z430_HUMAN,tr\|A0A5C2GLE7\|A0A5C2GLE7_HUMAN,tr\|A2A3R5\|A2A3R5_HUMAN,tr\|B2R6N6\|B2R6N6_HUMAN,tr\|B4DE78\|B4DE78_HUMAN,tr\|B4DFY5\|B4DFY5_HUMAN,tr\|B4DQY1\|B4DQY1_HUMAN,tr\|B4DTY8\|B4DTY8_HUMAN,tr\|B5BU24\|B5BU24_HUMAN,tr\|C9JIS1\|C9JIS1_HUMAN,tr\|E0X098\|E0X098_HUMAN,tr\|E7EUI6\|E7EUI6_HUMAN,tr\|E9PMD5\|E9PMD5_HUMAN,tr\|H0YIV0\|H0YIV0_HUMAN,tr\|Q6PYX1\|Q6PYX1_HUMAN,tr\|Q96HF4\|Q96HF4_HUMAN,tr\|V5YQL4\|V5YQL4_HUMAN |

**
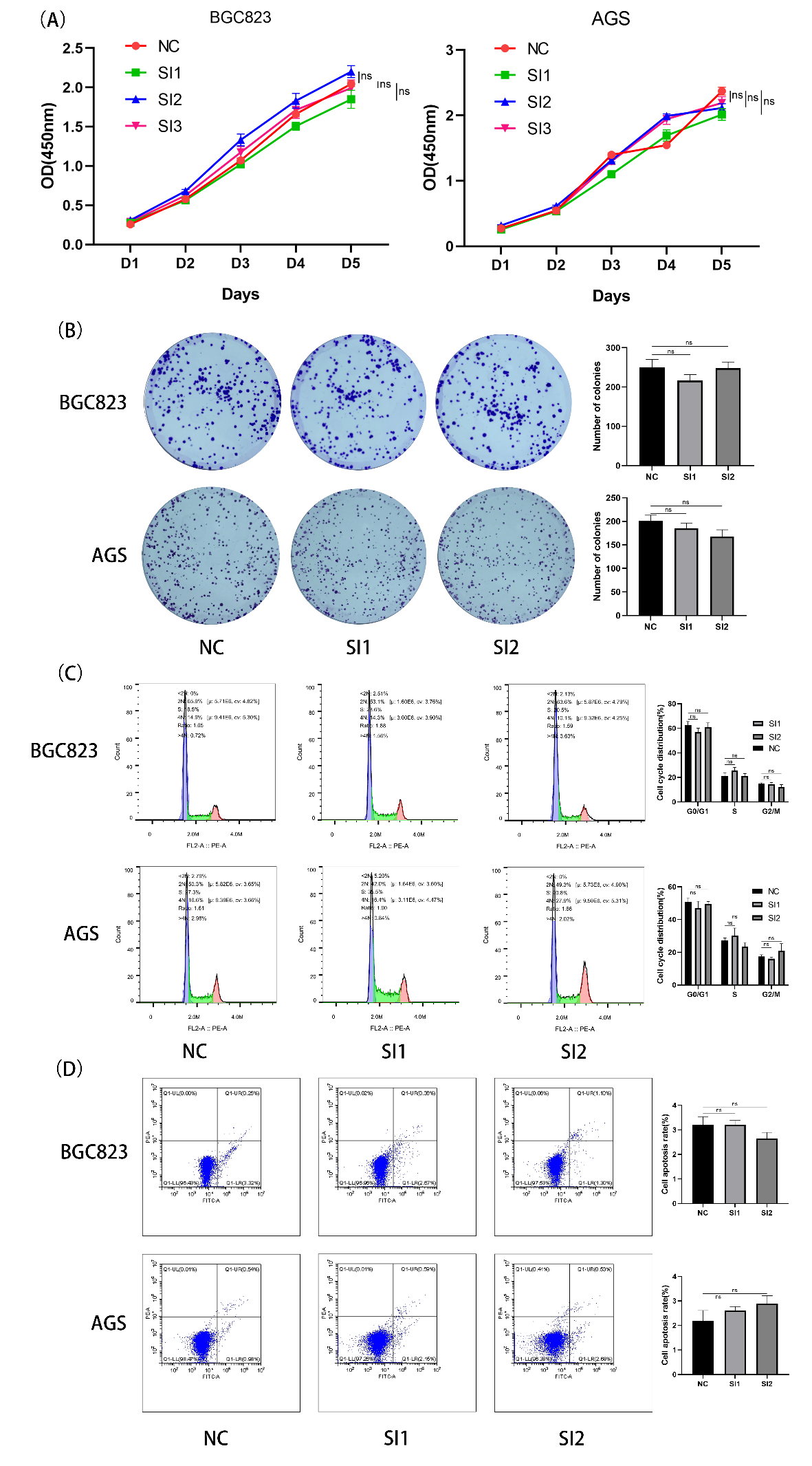
**

**Supplement figure 1 A, B. Knockdown of UBAP2L did not affect the viability of** **BGC823 and AGS measured with CCK-8 and colony formation assays. C, D. The changes of BGC823 and AGS cell cycle and apoptosis were detected by flow cytometry.**

**
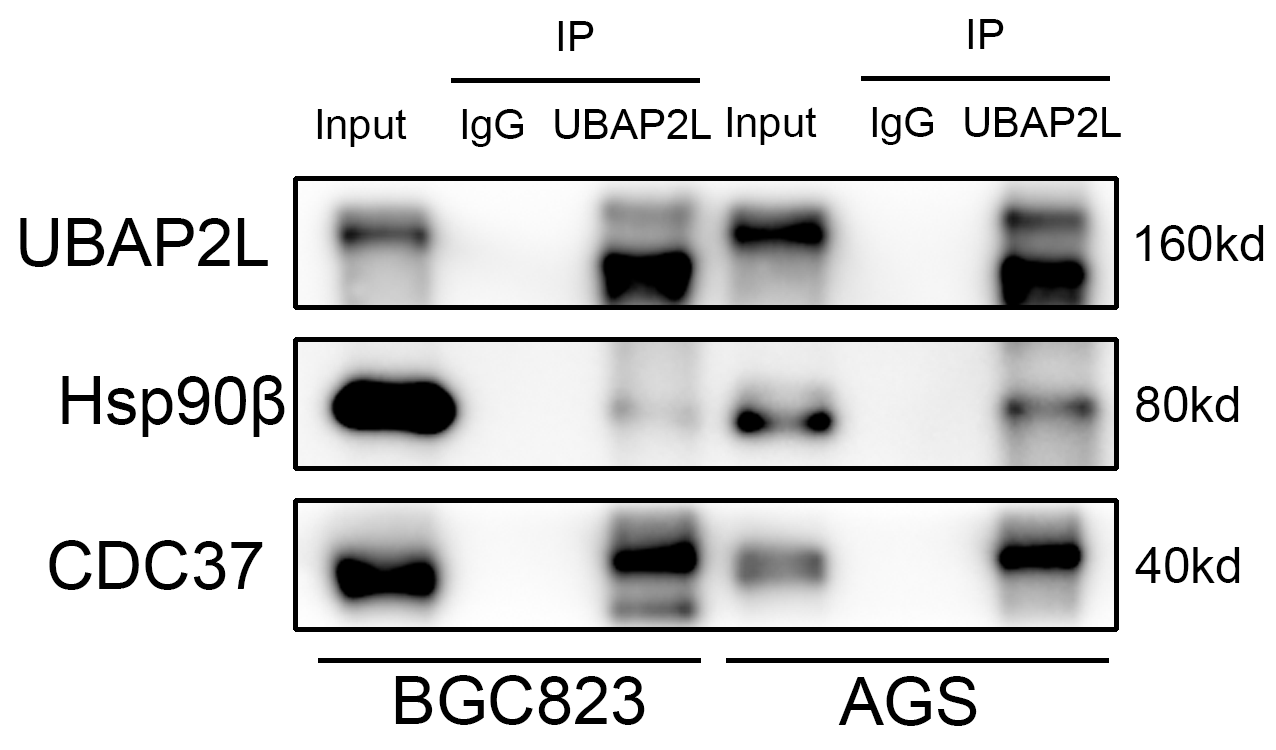
**

**Supplement figure 2 Western blot analysis of Co-IPs to demonstrate association of UBAP2L with Hsp90β and CDC37 in BGC823 and AGS cells.**
